# Supplementary material for: Non-destructive erosive wear monitoring of multi-layer coatings using AI-enabled differential split ring resonator based system
Source: Nat Commun. 2023 Aug 15;14:4916. doi: 10.1038/s41467-023-40636-9 (PMC10427693; doi:10.1038/s41467-023-40636-9)
Supplement: Supplementary file 1 — Supplementary Information [file 41467_2023_40636_MOESM1_ESM.pdf]

SUPPLEMENTARY INFORMATION

Non-Destructive Erosive Wear Monitoring of Multi-layer Coatings using AI-enabled Differential Split Ring Resonator based System

Vishal Balasubramanian[1], Omid Niksan[1], Mandeep C. Jain[1], Kevin Golovin[2], and Mohammad H. Zarifi[1,]*

[1] Okanagan MicroElectronics and Gigahertz Applications Laboratory, School of Engineering, Faculty of Applied Science, University of British Columbia, Kelowna, British Columbia, V1V 1V7, Canada.

[2] Department of Mechanical and Industrial Engineering, University of Toronto, Toronto, Ontario, M5S 3G8, Canada.

*Corresponding Author: Mohammad H. Zarifi, E-mail: mohammad.zarifi@ubc.ca

Table S1. Summary of the sensing operation, advantages, and limitations of various NDI sensors for erosion monitoring [1–10]

**NDI Sensing Modality**	**Working Principle**	**Advantages**	**Drawbacks**
Ultrasonic sensor [2,3]	Reflection of ultrasonic waves from Sample Under Test (SUT) surface	Multilayer corrosion detection; Large operational area	Cannot differentiate between corrosion surface and inner material surfaces; Needs media to couple pulses from the sensor to SUT; In-person monitoring
Terahertz (THz) sensor [4]	Reflection of 0.1-10 THz frequency waves from SUT surface	Good resolution; Can inspect inner layers; High Sensitivity	Cannot be used in moist environments
Eddy currents sensor [5–8]	Interaction between the magnetic field generated from a current-carrying coil and reflected from the SUT due to the eddy currents	Fast Operation; Inexpensive; Portable; Works on metals	Cannot detect coating detachment; Used mainly in metals
Acoustic emission sensor [1]	Uses transient wave released from strain energy present within the SUT surface	Useful for pits, cracks, and surface corrosion detection	Affected by environmental noises; Used for qualitative inspection
Magnetic particle inspection based sensor [9]	Magnetic flux leakage due to the presence of cavities or corrosion	High resolution; Quantitative inspection; High penetration depths; Multilayer erosion sensing; Portable	Suitable only for ferromagnetic material surfaces; Although portable, the sensor is relatively heavy
Thermo-graph sensors [10]	Temperature variations captured using infrared cameras to characterize SUT	Can monitor extensive surface areas in a short time	Expensive; Poor resolution on thick surfaces; Active thermography requires external heating system; Non-real-time sensing.

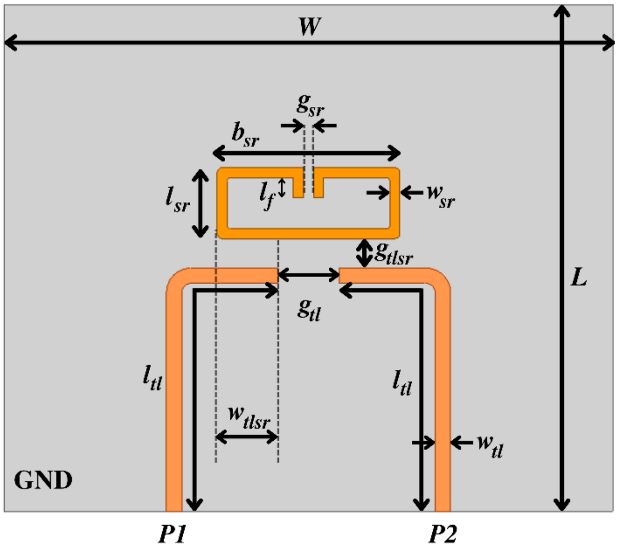

Fig S1. Design of the microwave split ring resonator-based non-destructive inspection system with the resonator placed on the top substrate, separate from the microstrip line on the bottom substrate. **Design Parameters:** *W*: 60 mm, *L*: 50 mm, g_{sr}: 1 mm, b_{sr}: 18 mm, l_{sr}: 7 mm, l_f: 2 mm, w_{sr}: 1 mm, g_{tl}: 6 mm, g_{tlsr}: 3 mm, w_{tlsr}: 6 mm, l_{tl}: 33 mm, w_{tl}: 1.5 mm. **Legend:** *GND:* Ground, *P1* and *P2*: Port 1 and Port 2

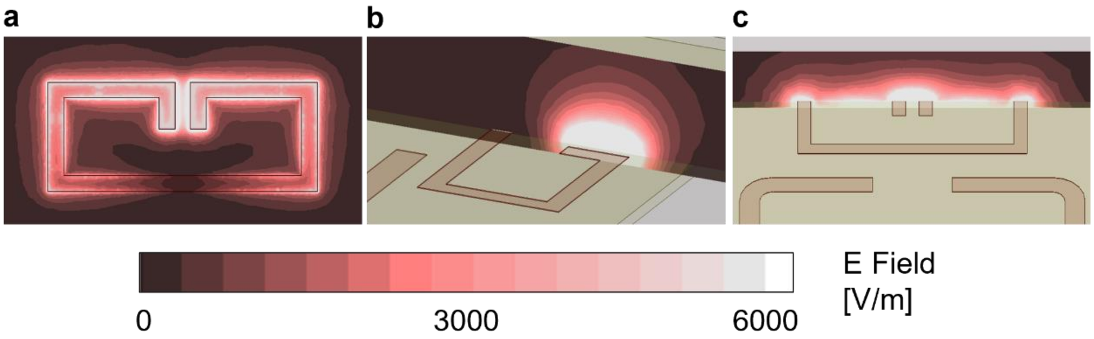

Fig S2. Electromagnetic field distribution around the split ring resonator when excited via the microstrip line **(a)** Surface view, **(b)** along X-axis, and **(c)** along Y-axis. The resonator was optimized for sensitive coating wear detection around the split ring gap indicated by the high electromagnetic field concentration of 6000 V/m at 2.5 GHz, verified by simulations using Ansys HFSS. Fig S2(b, c) depict the decreasing electromagnetic field concentration as the electromagnetic wave propagates away from the split ring gap, indicating the minimum material interaction at farther distances from the resonator leading to reduced sensitivity to erosive wear of thicker coatings.

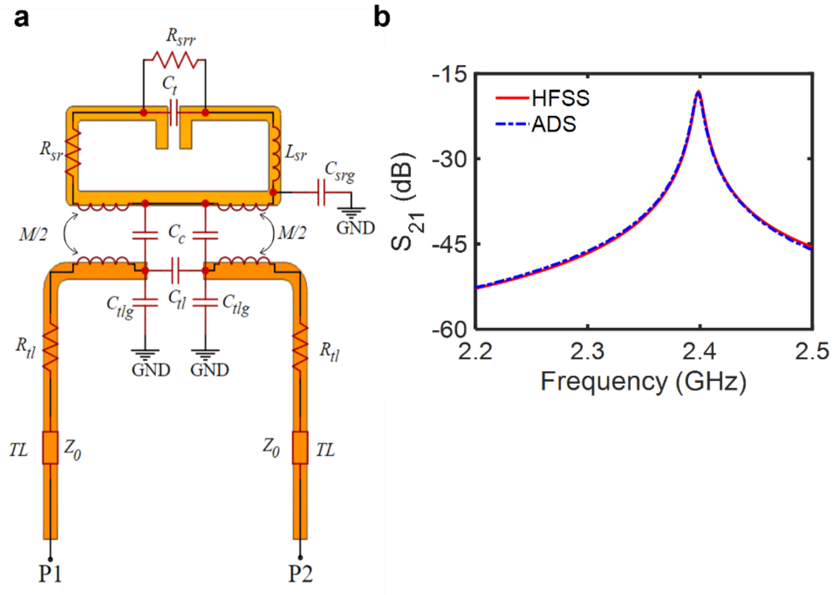

Fig S3. (a) Equivalent electrical circuit of the proposed system, and **(b)** Comparison of the simulated HFSS response and the lumped circuit model using ADS. **Design Parameters** (obtained through simulations using Advanced Design System) C_{srg}: 0.07 pF; C_c: 0.01 pF; C_{tl}: 0.035 pF; C_t: 0.05 pF; R_{tl}: 7.5 Ω; C_{tlg}: 0.65 pF; R_{sr}: 1.5 Ω; L_{sr}: 30 nH. **Legend:** *HFSS:* High-frequency structure simulator, *ADS:* Advanced Design System

The equivalent lumped circuit model analysis was performed for the developed system design for better understanding of the operation and response of the system to the erosive wear of the WRC. The circuit model and the parameters were designed and validated through simulations using Advanced Design System (ADS) and further confirmed through comparison with similar approximations performed in prior works[11,12]. The operation of the SRR was comparable to that of an LC resonator circuit with $f_r = 1/(2\pi\sqrt{LC_t})$, where L is the inductance due to the current loop path on the SRR, and C_t is the total capacitance in the SRR ring. Variations in the C_t value (a function of effective permittivity), led to changes in the resonant frequency while the inductance L was kept constant. When the system was not coated, the total capacitance C_t consisted of three parallel capacitances resulting in $C_t = C_{srr} + C_c + C_{srg}$. Here, C_{srr} is the intrinsic capacitance in the SRR, C_c is the coupling capacitance between the SRR and the MTL, and C_{srg} is the capacitance

formed between the SRR and the ground plane. However, when the system was coated with a WRC, the total capacitance increased by the value C_{wrc} (a function of relative permittivity and thickness of WRC) and thus, the resonant frequency varied as per Eq. S1.

$$f_r = \frac{1}{2\pi\sqrt{L(C_{srr}+C_c+C_{srg}+C_{wrc})}} \quad \text{(S1)}$$

Additionally, in the system model presented in Fig. 3, the introduction of the copper shields would introduce a capacitance parallel to the capacitances C_{srr} + C_c + C_{srg}, ultimately reducing the resonant frequency.

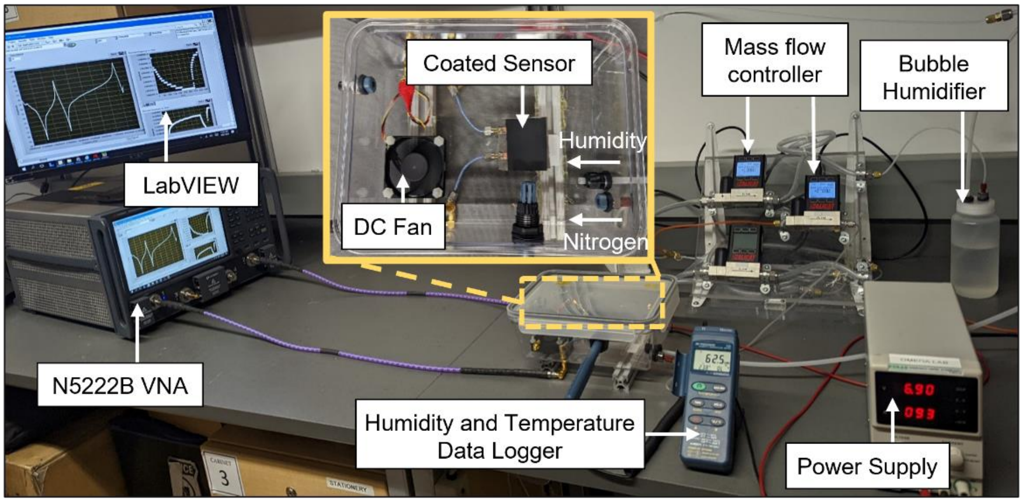

Fig S4. Experimental setup to measure the response of the coated microwave system against humidity variations. **Legend:** *DC:* Direct Current

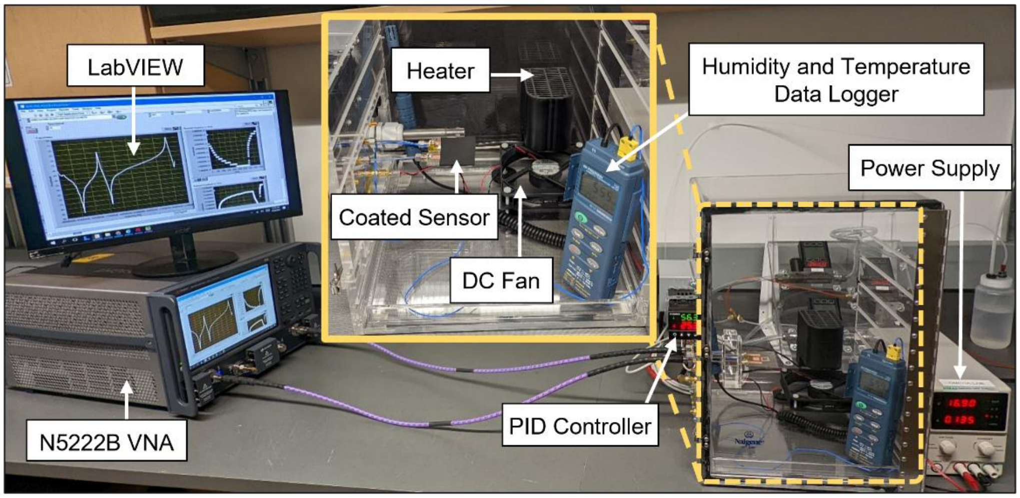

Fig S5. Experimental setup to measure the response of the coated microwave system against hot temperature variations. **Legend:** *DC:* Direct Current, *PID:* Proportional–Integral–Derivative

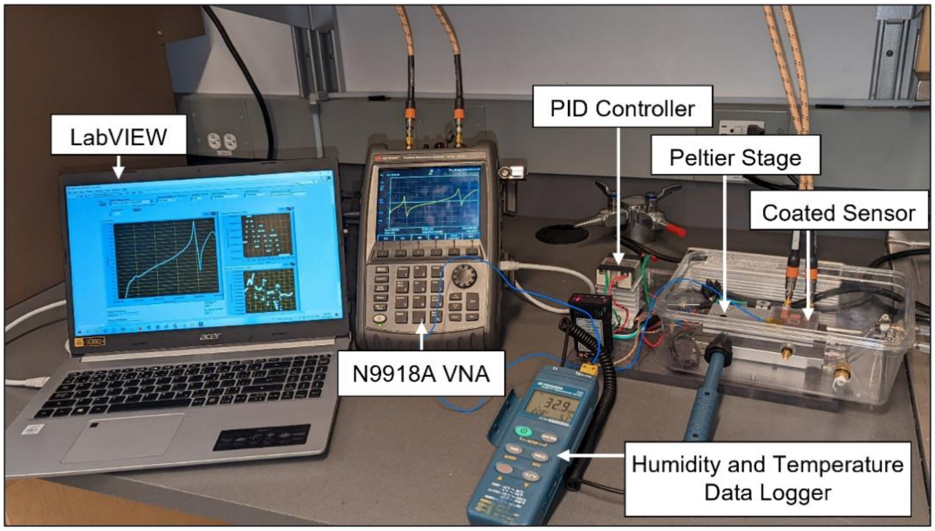

Fig S6. Experimental setup to measure the response of the coated microwave system against cold temperature variations. **Legend:** *PID:* Proportional–Integral–Derivative

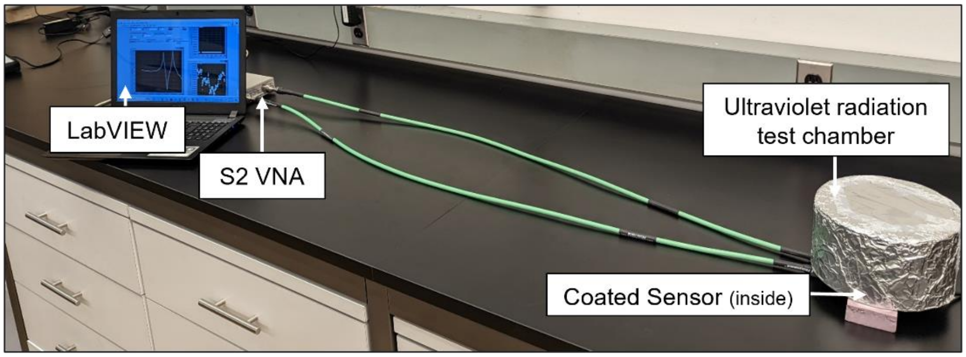

Fig S7. Experimental setup to measure the response of the coated microwave system against UV exposure.

REFERENCES

1. Zaki, A., Chai, H. K., Aggelis, D. G. & Alver, N. Non-Destructive Evaluation for Corrosion Monitoring in Concrete: A Review and Capability of Acoustic Emission Technique. *Sensors 2015, Vol. 15, Pages 19069-19101* **15**, 19069–19101 (2015).
2. Zhang, J. *et al.* Non-Destructive Evaluation of Coating Thickness Using Water Immersion Ultrasonic Testing. *Coatings 2021, Vol. 11, Page 1421* **11**, 1421 (2021).
3. Marcantonio, V., Monarca, D., Colantoni, A. & Cecchini, M. Ultrasonic waves for materials evaluation in fatigue, thermal and corrosion damage: A review. *Mech. Syst. Signal Process.* **120**, 32–42 (2019).
4. Cao, B., Wang, M., Li, X., Fan, M. & Tian, G. Noncontact Thickness Measurement of Multilayer Coatings on Metallic Substrate Using Pulsed Terahertz Technology. *IEEE Sens. J.* **20**, 3162–3171 (2020).
5. Raude, A., Europe, E., Bouchard, M. & Sirois, C. M. Stress Corrosion Cracking Direct Assessment of Carbon Steel Pipeline Using Advanced Eddy Current Array technology.
6. Grosso, M. *et al.* Eddy current and inspection of coatings for storage tanks. *J. Mater. Res. Technol.* **7**, 356–360 (2018).
7. Zhang, D., Yu, Y., Lai, C. & Tian, G. Thickness measurement of multi-layer conductive coatings using multifrequency eddy current techniques. *http://dx.doi.org/10.1080/10589759.2015.1081903* **31**, 191–208 (2015).
8. Wang, Z. & Yu, Y. Thickness and Conductivity Measurement of Multilayered Electricity-Conducting Coating by Pulsed Eddy Current Technique: Experimental Investigation. *IEEE Trans. Instrum. Meas.* **68**, 3166–3172 (2019).
9. Usarek, Z. & Warnke, K. Inspection of Gas Pipelines Using Magnetic Flux Leakage Technology. *Adv. Mater. Sci.* **17**, 37–45 (2017).
10. Maierhofer, C. *et al.* Application of impulse-thermography for non-destructive assessment of concrete structures. *Cem. Concr. Compos.* **28**, 393–401 (2006).
11. Rafi, M. A., Wiltshire, B. D. & Zarifi, M. H. Wideband Tunable Modified Split Ring Resonator Structure Using Liquid Metal and 3-D Printing. *IEEE Microw. Wirel. Components Lett.* **30**, 469–472 (2020).
12. Zarifi, M. H. *et al.* A microwave ring resonator sensor for early detection of breaches in pipeline coatings. *IEEE Trans. Ind. Electron.* **65**, 1626–1635 (2017).
