# Supplementary material for: Non-destructive erosive wear monitoring of multi-layer coatings using AI-enabled differential split ring resonator based system
Source: Nat Commun. 2023 Aug 15;14:4916. doi: 10.1038/s41467-023-40636-9 (PMC10427693; doi:10.1038/s41467-023-40636-9)
Supplement: Supplementary file 3 — Description of Additional Supplementary Files [file 41467_2023_40636_MOESM3_ESM.pdf]

Description of Additional Supplementary Files

File Name: Supplementary Code 1
Description: RNN model utilizing Keras with TensorFlow as described in the sub-section "Recurrent Neural Network Modeling".
